# Translational Issues with the Development of Cognition Enhancing Drugs

**DOI:** 10.3389/fneur.2014.00190

**Published:** 2014-09-25

**Authors:** Arjan Blokland, Nick van Goethem, Pim Heckman, Rudy Schreiber, Jos Prickaerts

**Affiliations:** ^1^Faculty of Psychology and Neuroscience, Department of Neuropsychology and Psychopharmacology, Maastricht University, Maastricht, Netherlands; ^2^Department of Psychiatry and Neuropsychology, School for Mental Health and Neuroscience, Maastricht University, Maastricht, Netherlands; ^3^Suadeo Drug Discovery Consulting LLC, Boston, MA, USA

**Keywords:** animal models, Alzheimer’s disease, cognition enhancing drugs, memory, pharmacology, pharmacokinetics, translational medical research, drug development

Franco and Cedazo-Minguez, in their review, raised the issue that animal models have a poor predictability with respect to drug development in the field of Alzheimer’s disease (AD) (1). Although this critical paper touches upon highly relevant issues in the development of new drugs, various other factors can be suggested, which also complicate the translation from animal studies to human studies. Here, we briefly discuss these issues relating to the development of cognition enhancers.

## Model and Test Validity: Do You Measure the Same?

A first issue is that the models that are being used in animal experiments have a poor validity. This relates to the deficit model that reflects a disease model, or the test model in which the behavior is interpreted in terms of learning and memory (2). There are various disease models for impaired learning and memory: pharmacological deficit models, lesion models, and genetic models [for review, see Ref. (3)]. Since these models can only mimic a specific aspect of a complex disease state, it is not surprising that these models can only have a limited value when new compounds are tested in these models. In addition, the use of test models puts another constraint on the translation of effects of drug. In human studies, episodic memory tests (in particular, verbal memory) or daily activities (ADL scales) are of special interest in assessing effects of drug on cognition. The face validity of animal test models (e.g., spatial Morris task, object memory) is obviously rather poor when comparing these memory capacities. In summary, it is clear that both disease models and test models have limited validity when translating effects of drug from animals to humans.

## Pharmacology: Dosing and Pharmacokinetics?

A second formidable challenge is to predict the efficacious dose of CNS drugs from animals to human (4). Several aspects are related to this point. CNS drugs typically show an inverted U-shaped dose–response curve, meaning that only a specific dose range leads to a beneficial effect on cognition. Especially, when the effective dose range is very narrow in animal studies, it will be difficult to titrate an effective dose for human studies. Moreover, side effects can be found at different doses across species with the consequence that the therapeutic window of drugs can be different for animals and humans. Another issue that could be mentioned in this context is related to the acute and chronic effects of drugs. Most animal studies evaluate the acute effects of cognition-enhancing drugs, whereas in human disease states, chronic treatments are considered to be more relevant. Eventually, chronic drug treatment is usually required for treating patients. However, chronic effects of drug may differ from acute effects of drugs. For instance, in healthy volunteers, a phosphodiesterase type 5 (PDE5) inhibitor was not effective on cognitive measures after acute treatment (5), although an effect on memory performance was found after chronic treatment with a PDE5 inhibitor (6). An additional point to be mentioned in this context is that the pharmacodynamics/pharmacokinetics for most drugs is different between species. For instance, PDE inhibitors have a short half-life in animals (7, 8), whereas in humans the half-life of PDE inhibitors is in general extended (9). In addition, the absorption and brain penetration are much faster in small experimental animals when compared to humans (10). Recently, we conducted a study in which we found significant brain penetration of PDE inhibitors within 10 minutes in rats after oral administration (unpublished data). This is much faster as can be expected in humans when considering species differences in plasma concentrations after drug administration. It is well known that psychopharmacological effects of drugs can be very different if the rate of absorption and brain penetration is fast or slow (11). Summarizing, there are many pharmacological factors for which animals and humans differ, which may explain differences in the effects on brain function. These are important points to consider when testing the effects of cognition-enhancing drugs in humans.

## Housing: Is Your Environment the Same?

A third and final general point we would like to raise in this commentary is related to the animals that are being used in most experimental studies. It has been suggested that standard housed animals may not be valid test models for the evaluation of cognition enhancing drugs (12). It was stated that due to a different brain development, standard housed animals could be considered as having an impoverished brain, and that this did not compare to normal brain development in humans [see also Ref. (13)]. Indeed, there are many differences between standard housed animals and animals raised in an enriched environment. Enriched animals outperform standard housed animals in many cognitive tasks, and the brain chemistry shows remarkable differences (14). Thus, enriched animals may resemble normal human brain development much better as compared to standard housed animals.

We recently conducted a study in which we tested the effects of a PDE5 inhibitor in enriched and standard housed animals (paper under review). Although we replicated our previous findings that acute treatment with PDE5 inhibitors improves memory performance in standard housed animals, we could not find a cognition enhancing effect of PDE5 inhibition in enriched animals. The lack of effect in enriched animals corroborate the human study with acute PDE5 treatment and suggest that standard housed animals may not be considered as relevant test models for cognition enhancing drugs.

## Conclusion

We fully agree with the considerations made by Franco and Cedazo-Minguez, yet it is our opinion that there are even other important points to consider when moving with a drug from animals to humans. Linked to this, we have brought forward some critical issues related to the development of cognition-enhancing drugs. Indeed, many challenges are still open to find an optimal strategy to translate animal data into optimal clinical studies to evaluate the cognition-enhancing potential of new drugs in humans.

## Conflict of Interest Statement

The authors declare that the research was conducted in the absence of any commercial or financial relationships that could be construed as a potential conflict of interest.
